# Supplementary material for: Residential Surrounding Greenspace and Mental Health in Three Spanish Areas
Source: Int J Environ Res Public Health. 2020 Aug 5;17(16):5670. doi: 10.3390/ijerph17165670 (PMC7460179; doi:10.3390/ijerph17165670)
Supplement: Supplementary file 1 [file ijerph-17-05670-s001.pdf]

Supplementary material

Residential Surrounding Greenspaceand Mental Health in Three Spanish Areas

Maria Torres Toda[1,2,3], Asier Anabitarte Riol[4,5],Marta Cirach[1,2,3], Marisa Estarlich[3,6,7], Ana Fernández-Somoano[3,8,9], Llúcia González-Safont[3,6],Mònica Guxens[1,2,3,10], Jordi Julvez[11,1,3],Isolina Riaño-Galán[3,9,12], Jordi Sunyer[1,2,3,13] and Payam Dadvand[1,2,3,*]

1 Barcelona Institute for Global Health (ISGlobal), 08003 Barcelona, Spain; maria.torres@isglobal.org (M.T.T.); marta.cirach@isglobal.org (M.C.); monica.guxens@isglobal.org (M.G.); jordi.sunyer@isglobal.org (J.S.); payam.dadvand@isglobal.org (P.D.)
2 Campus del Mar, Pompeu Fabra University (UPF), 08003 Barcelona, Spain
3 Spanish Consortium for Research on Epidemiology and Public Health (CIBERESP), 28029 Madrid, Spain; estarlich_mar@gva.es (M.E.); fernandezsana@uniovi.es (A.F.-S.); gonzalez_llu@gva.es (L.G.-S.); isogalan@yahoo.es (I.R.-G.)
4 Biodonostia Health Research Institute, Environmental Epidemiology and Child Development Group, 20014 San Sebastian, Spain; a-anabitarteriol@euskadi.eus (A.A.R.)
5 University of the Basque Country (UPV/EHU), Preventative Medicine and Public Health Department, Faculty of Medicine, 48940 Leioa, Spain
6 Epidemiology and Environmental Health Joint Research Unit, FISABIO-Universitat Jaume I-Universitat de València, 46010 Valencia, Spain
7 Department of Infirmary and Chiropody, Universitat de València, 46010 Valencia, Spain
8 Unit of Molecular Cancer Epidemiology, University Institute of Oncology of the Principality of Asturias (IUOPA) – Department of Medicine, University of Oviedo, 33006 Oviedo, Spain
9 Instituto de Investigación Sanitaria del Principado de Asturias (ISPA), 33011 Oviedo, Spain
10 Department of Child and Adolescent Psychiatry/Psychology, Erasmus University Medical Centre–Sophia Children's Hospital, 3015 Rotterdam, The Netherlands
11 Institut d'Investigació Sanitària Pere Virgili (IISPV), Hospital Universitari Sant Joan de Reus, 43204 Reus, Spain; jordi.julvez@isglobal.org (J.J.)
12 Servicio de Pediatría. Endocrinología, Hospital Universitario Central de Asturias (HUCA), 33011 Oviedo, Spain
13 Municipal Institute of Medical Research (IMIM-Hospital del Mar), 08003 Barcelona, Spain
* Correspondence: payam.dadvand@isglobal.org; Tel.: +34-93-214-73-29

Received: 15 June 2020; Accepted: 3 August 2020; Published: date

Table S1. Differences between excluded and included participants of the study.

Title	Excluded	Included	P value(b)
**Nº of participants n (%)**	1099 (57.6%)	1171 (42.4%)	
**Age mean (SD)**	33.8 (4.8)	34.8 (4)	0.00 **
**Ethnicity**			
White	1032 (93.9%)	896 (97.5%)	0.00 **
Others	67 (6%)	23 (2.5%)	
**Maternal education**			
Primary school	317 (31.4%)	258 (22.1%)	0.00**
Secondary school	401(39.7%)	490 (42%)	
University	291 (28.8%)	419 (35.9%)	
**Smoking**			
Yes	113 (26.7%)	315 (27.7%)	0.69
No	310 (73.3%)	821 (72.3%)	
**Maternal alcohol consumption**			
Yes	83 (9.8%)	112 (9.8%)	0.99

No	764 (90.2%)	1029 (90.2%)	
**Marital Status**			
Married	245 (88.1%)	909 (87.2%)	0.69
Others	33 (11.9%)	133 (12.8%)	
**IQ mean (SD)**	10.1 (3.0)	10.1 (3.0)	0.95
**Neighborhood Socioeconomic Status [(a)] mean (SD)**	0.5 (0.2)	0.5 (0.2)	0.10

[(a)]Urban Vulnerability Index. [(b)] Chi square test (categorical variables), Wilcoxon rank-sum test (continuous variables). p value<0.05**.

Table S2. Adjusted logistic regression models for each buffer of the average of residential surrounding greenspace at delivery and at 4-year follow-up, and risk of anxiety symptom dimension of the SCL-90-R. Odds Ratio (OR) and 95% confidence intervals (95% CI) for 1-IQR increase in each continuous indicator of residential surrounding greenspace.

Title	100m buffer	300m buffer	500m buffer
**Excluding single parents**			
Adjusted[(a)]	0.80 (0.56, 1.14)	0.67 (0.45,1.01)	0.65 (0.43,0.98)
**Excluding non-white participants**			
Adjusted	0.81 (0.58,1.13)	0.68 (0.46, 0.99) **	0.66 (0.44,0.97) **
**Adjusted for alcohol consumption during pregnancy**			
Adjusted	0.83 (0.59,1.16)	0.67 (0.45, 0.99)**	0.64 (0.43, 0.95) **
**Adjusted for smoking during pregnancy**			
Adjusted	0.83 (0.59,1.15)	0.68 (0.46, 1.00)	0.66 (0.44, 0.98) **
**Adjusted for second hand smoking**			
Adjusted	0.83 (0.60,1.16)	0.69 (0.47,1.02)	0.67 (0.45,0.99) **

[(a)]Adjusted for age, smoking, urban vulnerability index, educational attainment and IQ. p- value<0.05**.

Table S3. Adjusted and unadjusted logistic regression models for each buffer of residential surrounding greenspace at 4-year follow-up, and risk of each symptomatic dimension of the SCL-90-R. Odds Ratio (OR) and 95% confidence intervals (95% CI) for 1-IQR increase in each continuous indicator of residential surrounding greenspace.

Title	100m buffer	300m buffer	500 m buffer
**Global Severity Index**			
Unadjusted	0.92 (0.70,1.20)	0.84 (0.64,1.12)	0.85 (0.65,1.12)
Adjusted [(a)]	0.95 (0.70,1.29)	0.86 (0.62,1.20)	0.86 (0.62,1.19)
**Somatization**			
Unadjusted	0.70 (0.53,0.93) **	0.66 (0.49,0.89) **	0.69 (0.52,0.92) **
Adjusted	0.63 (0.45,0.89) **	0.62 (0.43,0.88) **	0.64 (0.45,0.92) **
**Obsessive-Compulsive**			
Unadjusted	0.94 (0.75,1.17)	0.85 (0.66,1.09)	0.83 (0.65,1.07)
Adjusted	0.95 (0.74,1.22)	0.84 (0.63,1.12)	0.82 (0.62,1.10)
**Interpersonal sensivity**			
Unadjusted	1.14 (0.93,1.39)	1.17 (0.93,1.46)	1.17 (0.93,1.46)
Adjusted	1.16 (0.92,1.46)	1.20 (0.93,1.56)	1.21 (0.93,1.57)
**Depression**			
Unadjusted	0.91 (0.68, 1.22)	0.87 (0.63,1.18)	0.84 (0.62,1.13)

Adjusted	0.93 (0.68, 1.27)	0.90 (0.64,1.26)	0.87 (0.62,1.22)
**Anxiety**			
Unadjusted	0.87 (0.68,1.13)	0.76 (0.57,1.03)	0.78 (0.58,1.04)
Adjusted	0.82 (0.59,1.13)	0.67 (0.46,0.98) **	0.68 (0.47,0.99) **
**Hostility**			
Unadjusted	1.01 (0.82,1.25)	0.95 (0.75,1.22)	0.90 (0.70,1.15)
Adjusted	1.06 (0.84,1.34)	0.98 (0.75,1.30)	0.92 (0.69,1.21)
**Phobic Anxiety**			
Unadjusted	0.87 (0.68,1.12)	0.92 (0.71,1.20)	0.98 (0.76,1.27)
Adjusted	0.91 (0.69,1.21)	0.98 (0.72,1.34)	1.04 (0.77,1.41)
**Paranoid Ideation**			
Unadjusted	1.02 (0.78,1.34)	1.00 (0.74,1.34)	1.01 (0.75,1.36)
Adjusted	1.04 (0.77,1.41)	1.05 (0.75,1.47)	1.05 (0.75,1.48)
**Psychoticism**			
Unadjusted	1.00 (0.81,1.23)	1.00 (0.79,1.26)	1.02 (0.81,1.28)
Adjusted	0.96 (0.75,1.23)	0.96 (0.72,1.27)	0.99 (0.75,1.30)

(a)Adjusted for age, smoking, urban vulnerability index, educational attainment and IQ. p- value<0.05 **.

Table S4. Adjusted and Unadjusted logistic regression models for each buffer of the average of residential surrounding greenspace at delivery and at 4-year follow-up, and risk of anxiety symptom dimension of the SCL-90-R. Odds Ratio (OR) and 95% confidence intervals (95% CI) for 1-IQR increase in each continuous indicator of residential surrounding greenspace stratified by age and by maternal educational attainment. .

Title	100m buffer	300m buffer	500m buffer
**Stratified by age**			
**Less than 35 years old**			
Unadjusted	0.93 (0.64,1.34)	0.83 (0.55,1.26)	0.80 (0.52,1.22)
Adjusted[(a)]	1.01 (0.66,1.53)	0.86 (0.53,1.39)	0.80 (0.49,1.32)
**More or equal to 35 years old**			
Unadjusted	0.82 (0.55,1.22)	0.72 (0.46,1.13)	0.73 (0.47,1.15)
Adjusted[(a)]	0.65 (0.36,1.15)	0.53 (0.27,1.02)	0.55 (0.29,1.05)
**Stratified by educational attainment**			
**Primary school or lower**			
Unadjusted	1.07 (0.62,1.87)	0.94 (0.49,1.79)	0.84 (0.43,1.63)
Adjusted[(b)]	0.83 (0.40,1.74)	0.84 (0.39,1.79)	0.53 (0.21,1.33)
**High school**			
Unadjusted	0.70 (0.39,1.23)	0.56 (0.29,1.06)	0.59 (0.31,1.12)
Adjusted[(b)]	0.81 (0.46,1.43)	0.61 (0.32,1.18)	0.67 (0.34,1.32)
**University or higher**			
Unadjusted	1.30 (0.72,2.34)	1.16 (0.58,2.31)	1.13 (0.54,2.37)
Adjusted[(b)]	1.22 (0.55,2.71)	0.92 (0.42,2.01)	0.86 (0.31,2.33)

(a)Adjusted for smoking, urban vulnerability index, educational attainment and IQ. (b)Adjusted for age, smoking, urban vulnerability index and IQ.
